# Case report: A pregnant woman with recurrent craniopharyngioma: surgical decision-making and doctor-patient bond

**DOI:** 10.3389/fonc.2024.1508803

**Published:** 2024-12-23

**Authors:** Ao Shen, Yue Min, Shuanghong He, Dongjie Zhou, Shu Jiang, Peizhi Zhou

**Affiliations:** ^1^ Department of Neurosurgery, West China Hospital/West China School of Medicine, Sichuan University, Chengdu, Sichuan, China; ^2^ Department of Neurosurgery, West China Hospital/West China School of Nursing, Chengdu, Sichuan, China; ^3^ Health Management Center, West China Hospital/West China School of Nursing, Chengdu, Sichuan, China

**Keywords:** recurrent craniopharyngioma, pregnancy, surgical strategy, hypothalamic syndrome, hormone replacement therapy

## Abstract

Craniopharyngioma is a rare, benign tumor that originates from the pituitary stalk and extends along the pituitary-hypothalamic axis. It can have serious effects due to its location, affecting hormone regulation, vision, and other neurological functions. It is particularly rare and challenging to manage it during pregnancy due to the potential impacts on both maternal and fetal health, requiring careful, individualized treatment. We reported a 26-year-old pregnant woman at 27 weeks with recurrent craniopharyngioma who presented with worsening consciousness and hydrocephalus. Despite recommendations to terminate the pregnancy for tumor resection, she chose to continue. We performed an endoscopic endonasal tumor resection with pituitary transposition to preserve pituitary function, and at 32 weeks, she delivered a healthy baby via cesarean section. We provided a detailed account of the perioperative complications and their management, addressing endocrine, temperature, and fluid regulation challenges. Throughout the process, the medical team maintained open communication with the patient and her family, respecting their desire to continue the pregnancy and exemplifying patient-centered, compassionate care.

## 1 Introduction

Craniopharyngioma is a rare neoplasm originating from vestigial remnants of the craniopharyngeal anlage, typically characterized by cysts lined with stratified squamous epithelium. These tumors account for approximately 1.2% to 4.6% of all intracranial tumors, with an incidence of 0.5 to 2.5 new cases per million people annually worldwide (1, 2). Manifestation during pregnancy is exceptionally rare (3–9). Histologically, neurons from the hypothalamus extend into the neurohypophysis, forming the hypothalamic-pituitary axis, with the pituitary stalk and neurohypophysis serving as extensions of the hypothalamus. These structures contain axons that transport vasopressin and oxytocin from the supraoptic and paraventricular nuclei to the posterior pituitary. Endocrine dysfunction of the anterior pituitary is reported in 35% to 100% of craniopharyngioma patients, while posterior pituitary dysfunction occurs in 6% to 38% (10–12).

We present the case of a 26-year-old woman, 27 weeks pregnant, admitted with significant disturbance of consciousness caused by a recurrent craniopharyngioma. The patient, standing 156 cm tall and weighing 54 kg, was found to be drowsy but responsive to verbal stimuli, though she struggled to answer questions. Neurological examination revealed a left pupil of 2 mm and a right pupil of 3 mm, both with sluggish light reflexes. Severe visual impairment was noted in the left eye, with only light perception remaining, and no pathological reflexes were present. Despite the recurrence during pregnancy, the patient chose to continue her pregnancy. Following multidisciplinary team (MDT) evaluation, an endoscopic endonasal tumor resection with pituitary transposition was performed to preserve pituitary function. Postoperatively, the MDT effectively managed complications, including persistent fever, pulmonary edema, diabetes insipidus, and endocrine dysfunction. At 32 weeks, she delivered a healthy baby girl via cesarean section, and both mother and child were discharged in stable condition.

This case provides a detailed account of perioperative complications and management strategies. Given the rarity of craniopharyngioma and the even rarer occurrence of successful surgical resection of recurrent craniopharyngioma during pregnancy followed by successful childbirth, this case underscores the importance of precision medicine, individualized management, and compassionate care to ensure the safety of both mother and child.

## 2 Case description

**Figure 1:**
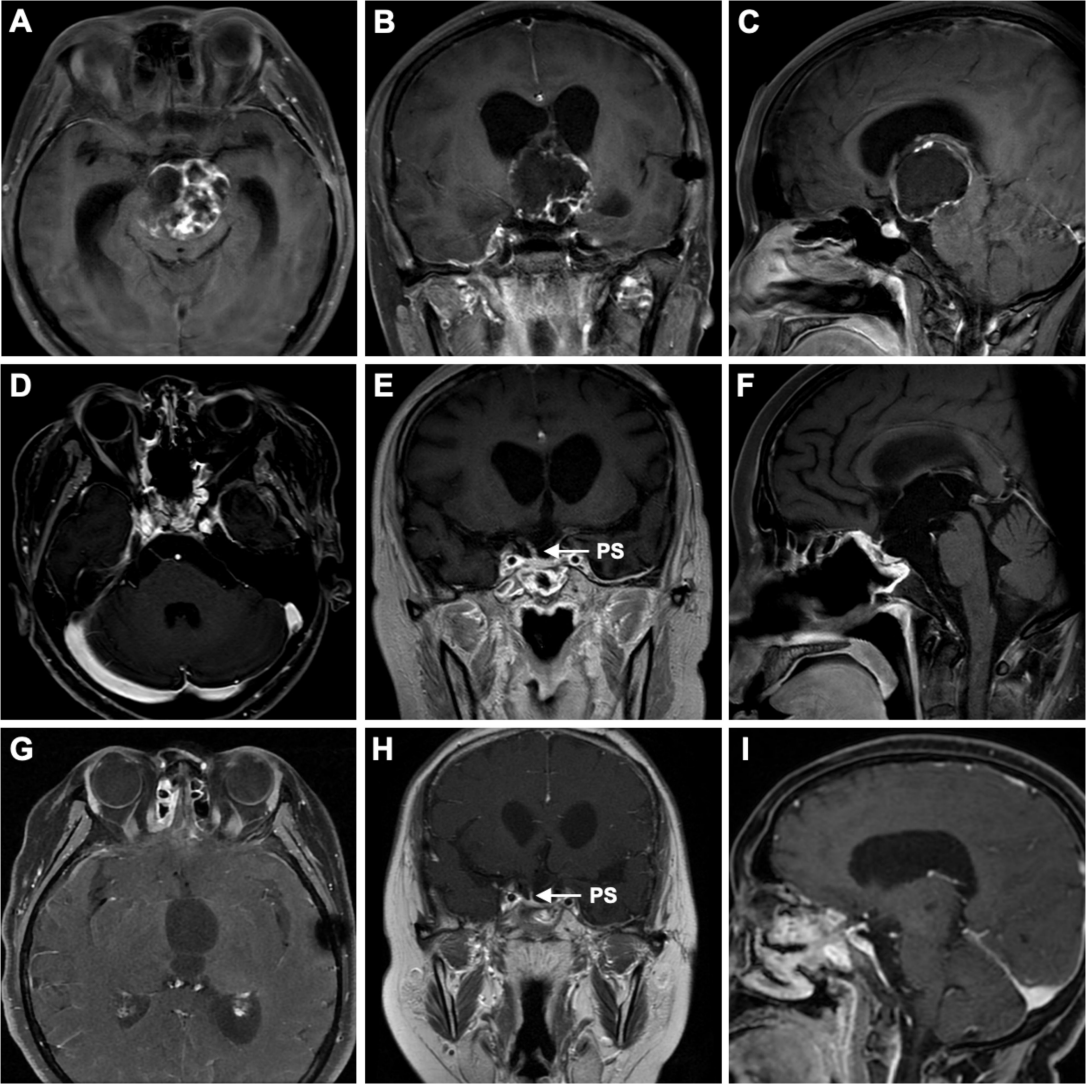
Preoperative correlative axial **(A)**, coronal **(B)**, and sagittal **(C)** T1-weighted post–contrast-enhanced MRI revealed a suprasellar mass with a cystic-solid composition, occupying the entire third ventricle and interpeduncular cistern. The lesion severely compressed structures such as the hypothalamus and the foramen of Monro, leading to lateral ventricular enlargement and resulting in hydrocephalus. Three-month postoperative correlative axial **(D)**, coronal **(E)**, and sagittal **(F)** T1-weighted post-contrast-enhanced MRI showed gross total resection (GTR) of the tumor, with preservation of the pituitary stalk. Six-month postoperative correlative axial **(G)**, coronal **(H)**, and sagittal **(I)** T1-weighted post-contrast-enhanced MRI showed no signs of tumor recurrence and also demonstrated the preserved pituitary stalk. PS, pituitary stalk.

A 26-year-old female, at 27 weeks of pregnancy, was admitted to the neurosurgery department of our hospital due to one month of drowsiness, which had worsened into a significant disturbance of consciousness over the past day. At 23 weeks of pregnancy, she had previously visited a large neurosurgery center because of altered consciousness. Eleven years earlier, the patient had undergone craniotomy at the same center for a suprasellar mass, which was confirmed postoperatively to be a craniopharyngioma. Since the surgery, she had been on long-term hormone replacement therapy (hydrocortisone and levothyroxine) due to postoperative hypopituitarism and had intermittently used desmopressin (Minirin) to manage diabetes insipidus (DI), but she was unable to maintain regular follow-up due to several reasons. After a thorough examination, the doctors suspected a recurrence of craniopharyngioma. Given her pregnancy and weakened physical condition, the doctors recommended termination of the pregnancy, followed by tumor resection. However, the patient and her family declined, as she was determined to continue the pregnancy. Over the next few weeks, her consciousness rapidly deteriorated, and she developed a persistent high fever, leading to her admission to our neurosurgery department. On the second day of admission, a contrast-enhanced MRI of the brain revealed a 4.5 × 3.8 cm round cystic-solid mass in the sellar region. Enlargement of the sellar was observed, along with significant compression of the third ventricle and interpeduncular cistern, leading to obstructive hydrocephalus (**Figures 1A–C**).

We quickly assembled an MDT including anesthesiology, obstetrics, endocrinology, and Neurointensive Care Unit (NICU) to formulate a treatment plan. The intracranial mass’s compression of the third ventricle, hypothalamus, brainstem, and small perforating vessels posed a high risk of postoperative complications, such as DI, hypopituitarism, and hypothalamic syndrome. The patient’s previous craniotomy of craniopharyngioma 11 years ago increased surgical risk due to potential adhesions. Additionally, the stress, hormonal fluctuations, and physical demands of pregnancy further compromised her health, making tumor surgery during pregnancy significantly riskier than other brain surgeries. The MDT initially recommended prioritizing the mother’s safety by performing tumor resection and terminating the pregnancy simultaneously. However, after thorough discussions with the patient and her family, their strong desire to continue the pregnancy led us to reconsider our plan. Ultimately, we chose to give both the mother and the unborn child a chance: proceed with tumor removal while continuing the pregnancy, and reassess the need for pregnancy termination based on the postoperative outcome.

**Figure 2:**
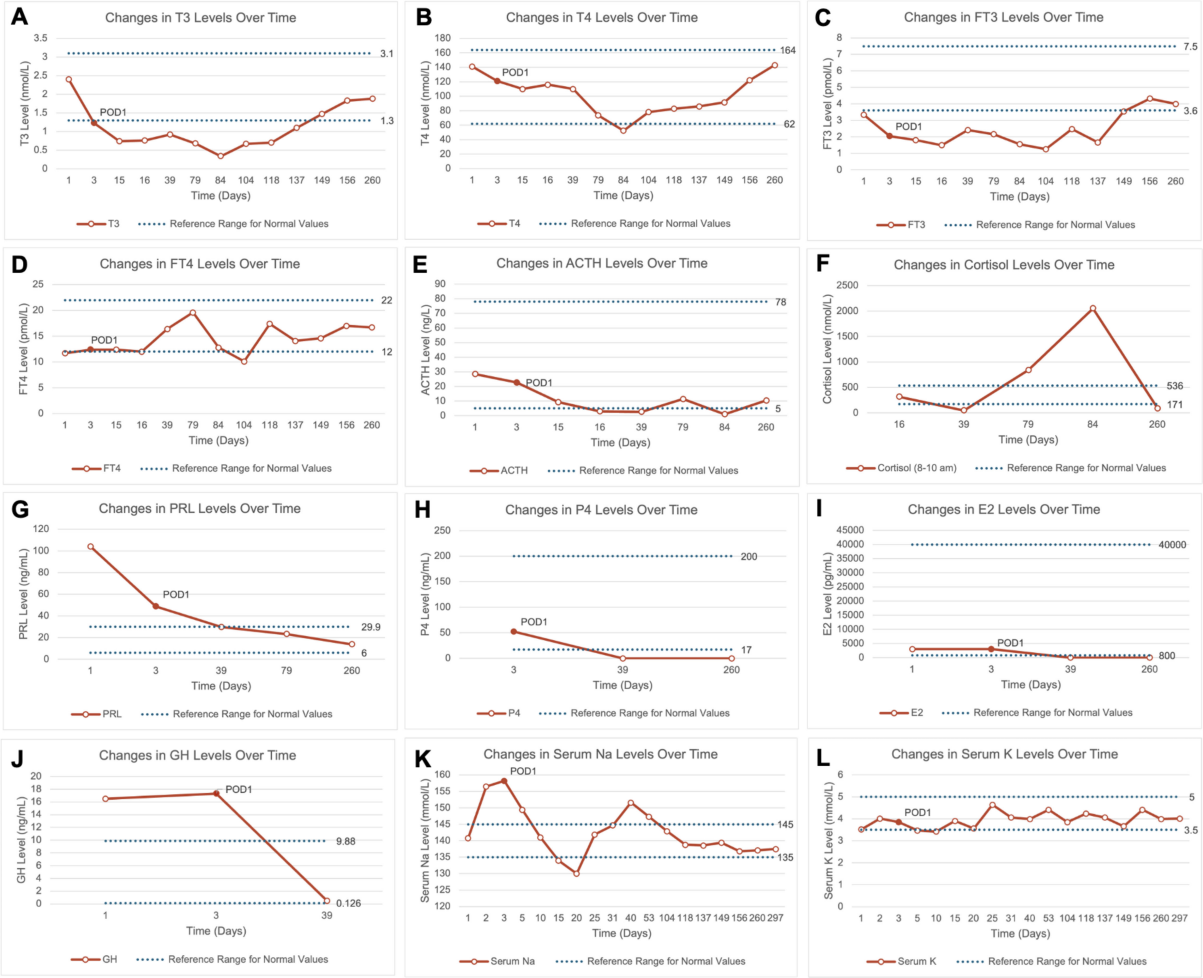
Line charts showing changes in pituitary hormones, sex hormones, and serum electrolytes over time preoperatively and postoperatively. **(A–J)** Changes in T3, T4, FT3, FT4, ACTH, cortisol, prolactin, progesterone, estradiol, and growth hormone levels over time preoperatively and postoperatively. The final recorded time point is day 260 post-admission, showing that most hormone levels remained within normal ranges; **(K, L)** Changes in serum sodium and potassium levels over time preoperatively and postoperatively. The final recorded time point is day 297 post-admission, and results were within normal ranges. ACTH, Adrenocorticotropic Hormone; E2, Estradiol; FT3, Free Triiodothyronine; FT4, Free Thyroxine; GH, Growth Hormone; P4, Progesterone; POD1, Postoperative Day 1; PRL, Prolactin; T3, Triiodothyronine; T4, Thyroxine.

Thus, after the patient was admitted to our hospital in an emergency on August 31, 2023, a head MRI, MDT consultation, and preoperative preparation were completed the following day. On the morning of the third day, she was taken to the operating room for surgery. Under intraoperative neurophysiological monitoring, we performed an endoscopic endonasal tumor resection utilizing the pituitary stalk transposition technique. This approach primarily involves a partial incision of the dura mater to achieve adequate mobilization of the pituitary gland and its superior and inferior arteries, while preserving the dura. Only one side of the inferior hypophyseal artery was severed, allowing full exposure of the retrosellar and interpeduncular cistern for tumor removal. By releasing and rotating the pituitary gland and stalk by nearly 90°, we shifted the tumor’s point of origin relative to the residual pituitary stalk from an anterior-posterior orientation to a lateral one. This repositioning provided a direct surgical angle for sharp dissection of the tumor from the residual stalk. The technique preserves at least one side of the superior hypophyseal artery (SHA), one side of the inferior hypophyseal artery, and the hypophyseal portal system on the anterior surface of the stalk, thereby maximizing preservation of pituitary function and venous drainage (13–15). The surgery was successful, and the patient was transferred to the NICU for further treatment. She was treated with third-generation cephalosporin 2g q8h for infection control (WBC 10.82×109/L, PCT 0.37 ng/ml, IL-6 20 pg/ml); hydrocortisone 200 mg qd was continuously infused (ACTH 22.65 ng/L), and 1-deamino-8-d-arginine vasopressin (DDAVP) 2 µg q12h was administered to manage DI (serum sodium 158.2 mmol/L, potassium 3.85 mmol/L) (**Figure 2**). Six hours after surgery, the patient responded to verbal stimuli and partially followed commands, with a Glasgow Coma Scale (GCS) score of E4VTM5. On the second postoperative day, she was successfully extubated and was placed on 3L/min of oxygen via face mask, maintaining 100% oxygen saturation. Fetal monitoring remained stable and unchanged from preoperative levels. However, in the afternoon, the patient experienced a progressive decline in oxygen saturation, with auscultation revealing extensive crackles in both lungs. Arterial blood gas analysis indicated type I respiratory failure, necessitating reintubation. Bedside lung ultrasound showed B-lines in both lungs, suggestive of diffuse pulmonary edema (**Supplementary Video S1**). An MDT, including specialists from neurosurgery, NICU, pulmonology, obstetrics, endocrinology, infectious disease, and the pharmacy department, was convened again for further discussion: 1) Pulmonary edema was most likely due to fluid overload, although central causes could not be excluded. Fluid management was adjusted, DDAVP was continued to maintain water-electrolyte balance, and Torsemide (with minimal fetal impact) was added if necessary. 2) The patient’s postoperative fever was unlikely to be caused by infection, as multiple lumbar punctures (performed on September 3, 5, and 11) showed no evidence of intracranial infection (biochemical analysis, cytology, bacterial and fungal cultures, G-test, GM-test, and NGS). Considering the risk of pneumonia following pulmonary edema, third-generation cephalosporin were administered to cover the possibility of a pulmonary infection. 3) Hydrocortisone replacement therapy was continued, with plans to taper as the patient stabilized. 4) Fetal heart rate remained elevated, raising concerns about fetal distress and hypoxia. The patient and her family were informed, and preparations were made for a potential emergency cesarean section to deliver a premature infant.

After managing a series of postoperative complications, including hypothalamic syndrome, fever, and DI, the patient was successfully extubated on September 11 and transitioned to non-invasive ventilation. Due to her hypercoagulable state during pregnancy, low-molecular-weight heparin anticoagulation was initiated on September 12, and third-generation cephalosporin was discontinued on September 14. During this period, central hyperthermia was managed with cooling blankets. Regular monitoring, including bedside critical care ultrasound, fetal heart monitoring, obstetric ultrasound, pituitary hormone levels, and electrolytes, was conducted. On September 28, at 32 weeks of gestation, an obstetric ultrasound revealed oligohydramnios, meeting the criteria for emergency cesarean delivery. A 1.9 kg female infant with an Apgar score of 9-10-10 was delivered. The patient was transferred to the NICU for postoperative care, extubated on September 29, and her temperature gradually normalized. By October 8, cooling blankets were discontinued, and she was transferred to the general ward fully conscious. The patient was discharged on October 14, 2023.

During follow-up, the newborn showed healthy growth, while the mother was hospitalized once due to sepsis caused by an E. coli infection but was eventually discharged in stable condition. The postoperative pathological diagnosis was adamantinomatous craniopharyngioma. Immunohistochemical staining showed P63 (+), CK5/6 (+), and Beta-Catenin (partial +). Genetic testing detected a missense mutation in codon 35 of exon 3 of the CTNNB1 gene (c.104T>A, 105C>A; p.Ile35Lys), while no point mutation was found in exon 15 of the BRAF gene (V600E). During follow-up, there were no signs of craniopharyngioma recurrence (**Figures 1D–I**). Meanwhile, the patient’s anterior pituitary function gradually returned to normal range during follow-up. After the cesarean section, estradiol and progesterone levels also returned to normal, and serum sodium and potassium levels fluctuated within normal ranges as well (**Figure 2**).

## 3 Discussion

### 3.1 Historical case insights and management

Craniopharyngioma in pregnancy presents a unique challenge due to its rarity and the complexities it introduces to both maternal and fetal health. Over the decades, a limited number of cases have been documented, each providing valuable insights into management strategies. From early surgical interventions to hormone therapies, these cases highlight the delicate balance required to ensure successful outcomes for both mother and child. Despite this growing body of literature, our case introduces a novel approach, emphasizing the successful integration of multidisciplinary care and advanced surgical techniques. This report not only adds to the limited pool of data but also underscores the innovation and individualized treatment required in such rare and complex cases. Fischer et al. described two cases of craniopharyngioma in pregnancy with visual impairment, one delivering at 34 weeks and the other opting for therapeutic abortion (16). Corral et al. reported a woman who conceived through ovulation induction after craniopharyngioma resection and delivered at 37 weeks (17). Sachs et al. documented a case where complete tumor resection at 29 weeks resulted in full-term vaginal delivery (18). Van der Wildt et al. described a woman diagnosed with DI during pregnancy who delivered at 36 weeks and had successful tumor resection postpartum (19). Kodama et al. and Hiett et al. reported similar cases of women with craniopharyngioma, both delivering preterm and undergoing successful tumor resection postpartum (5, 8). Johnson et al. described a woman whose vision was restored after tumor resection in the second trimester, delivering a healthy baby at term (6). Begon et al. reported seven women treated with GnRH after surgery, all achieving ovulatory cycles (20). Maniker et al. and Aydin et al. both described cases of craniopharyngioma recurrence during pregnancy, with successful transsphenoidal surgery and term deliveries (9, 21). Overton et al. reported three cases of craniopharyngioma patients delivering via cesarean after ovulation induction (22). Hayashi et al. and Volz et al. both described women with panhypopituitarism successfully delivering after fertility treatments (23, 24). Kübler et al. reported a woman delivering at 38 + 4 weeks after hormone replacement therapy (25). Zoia et al. described a case involving cesarean delivery at 33 weeks followed by successful tumor resection (7).

### 3.2 Surgical decision-making and technique

In our case, selecting the surgical plan was a complex and challenging process requiring careful consideration. Given the patient’s pregnancy and the tumor’s complexity, the MDT reviewed several options, each with its own benefits and risks. One option was lateral ventricular puncture or ventriculoperitoneal shunt placement to temporarily relieve intracranial pressure, allowing more time for the pregnancy to reach full term before performing tumor resection. However, the need for an abdominal incision during pregnancy increased the risk of infection and complications, making this approach less feasible. Another option involved placing an Omaya reservoir to periodically drain the tumor fluid, reducing its size and alleviating hydrocephalus. While this option carried lower surgical risks and allowed more time for pregnancy continuation, rapid deterioration might still necessitate early termination and immediate tumor resection. A third option was craniotomy or endoscopic transnasal tumor removal, intended to relieve pressure on critical structures, thus reducing the risk of coma and hypothalamic complications. However, this approach would involve terminating the pregnancy, requiring close collaboration with obstetricians to ensure maternal and neonatal safety. The most challenging decision was to proceed with tumor removal while continuing the pregnancy. Although this option carried higher risks, it respected the patient’s and her family’s strong desire to maintain the pregnancy. Ultimately, after carefully weighing surgical safety, maternal and fetal health risks, and the patient’s wishes, we decided to proceed with tumor removal while maintaining the pregnancy. This decision was based on medical feasibility and a deep respect for the patient’s autonomy and a commitment to compassionate care.

The surgical approach we employed is the endoscopic interdural transcavernous pituitary release and rotation technique. This procedure involves removing the ventral outer layer of dura surrounding the pituitary gland and half of the dorsal aspect, including one side of the diaphragm sellae, dorsum clival dura, and the superior wall of the cavernous sinus. This provides full access to remove tumors like craniopharyngioma in the retrosellar space and interpeduncular cistern. By releasing and rotating the pituitary gland and stalk nearly 90°, we can dissect the tumor from the depressed residual stalk and the attached hypophyseal portal system at an optimal angle. This technique preserves at least one side of the superior hypophyseal artery, inferior hypophyseal artery, and the related hypophyseal portal system, which is critical for maintaining pituitary function in this pregnant patient.

### 3.3 Fluid management strategy

The fluid management strategy for pregnant women with craniopharyngioma requires a careful balance of fluid control, medication, and monitoring to ensure the safety of both mother and fetus. During pregnancy, maternal blood volume increases, cardiac output rises, and the heart enters a high-output state, increasing the risk of pulmonary edema and reliance on positive pressure ventilation. Managing fluids must not only reduce pulmonary overload but also maintain adequate placental blood flow and fetal circulation, making it a complex process that demands MDT involvement. The choice of diuretics is critical, as pregnant women are more sensitive to certain medications. Diuretics like torasemide, which have minimal fetal impact, are preferred to reduce maternal pulmonary edema. Desmopressin, commonly used in craniopharyngioma patients who often experience postoperative diabetes insipidus and hypernatremia, must be used cautiously. Overuse can disrupt electrolyte balance, requiring strict monitoring of electrolyte levels and urine output, with individualized dosage adjustments as necessary. The type of fluids administered is also important. Colloid-based solutions, such as albumin, help increase colloid osmotic pressure, reducing pulmonary edema while stabilizing maternal circulation. This approach prevents excessive fluid depletion, which could negatively impact fetal development. In addition to maintaining maternal blood volume, close monitoring of both maternal and fetal circulation is vital. Critical care ultrasound can assess maternal cardiac and pulmonary function, while regular checks of fetal heart rate and amniotic fluid ensure fetal safety throughout treatment (**Supplementary Figure S1**).

### 3.4 Temperature management

**Figure 3:**
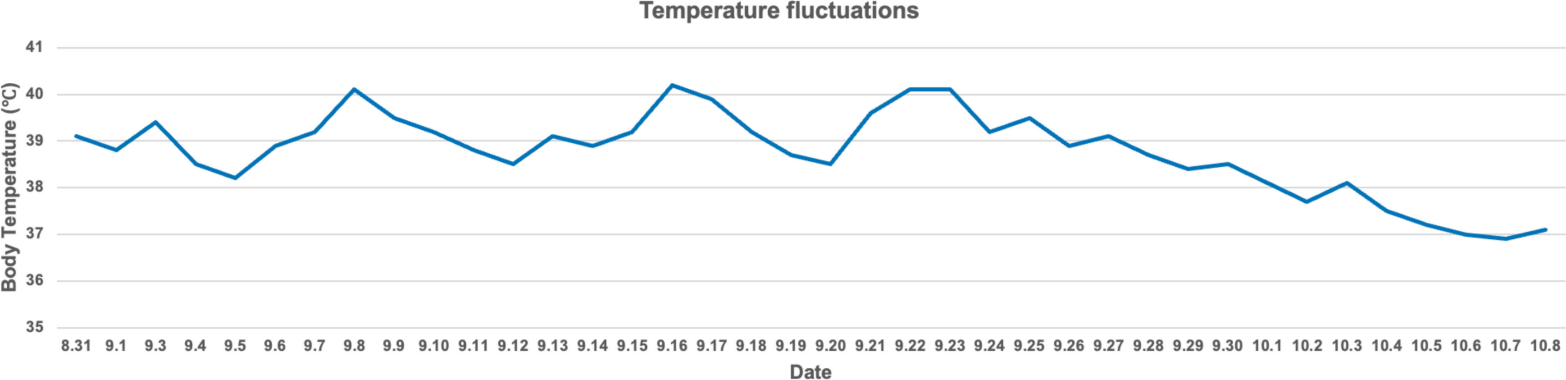
The body temperature fluctuations of this patient. Notably, significant temperature spikes occurred on September 8, 16, and 22. These spikes were effectively managed using a cooling blanket, resulting in a successful reduction in body temperature to safer levels. Over time, the patient’s body temperature became progressively stable, with a noticeable decline starting in late September. By early October, the temperature had nearly returned to normal levels.

The patient’s temperature management involved several complex fluctuations, starting with preoperative high fever, brief postoperative recovery, recurrent fever, and eventual stabilization through the use of a cooling blanket. Preoperatively, the patient’s temperature reached nearly 39°C. Though it temporarily normalized after surgery, it spiked again with significant fluctuations. The cooling blanket effectively controlled the fever, but recurrences were noted on September 8, 16, and 22, with each time successfully managed with the same method. After a cesarean section on September 28, the patient’s temperature stabilized, and by October 6, after discontinuing the cooling blanket, it fully normalized (**Figure 3**). This process highlights the importance of early detection and intervention in fever management. While the temperature briefly stabilized postoperatively, the rapid recurrences emphasized the need for close monitoring during the early postoperative period, likely due to central dysregulation. The cooling blanket proved essential for controlling hyperthermia, offering a non-invasive method to quickly reduce fever and prevent central nervous system damage. The absence of an infection source, as confirmed by lab tests, suggested the fever was central in origin rather than infectious. Dynamic monitoring of infection markers like interleukin-6 and procalcitonin allowed timely adjustments to antibiotic therapy, preventing unnecessary side effects. An MDT comprising neurosurgery, critical care, and infectious disease specialists played a crucial role. The MDT carefully assessed each fever spike and adjusted the treatment strategy, improving the efficiency of temperature management and supporting the patient’s overall recovery.

### 3.5 Hormone replacement therapy

In pregnant women with craniopharyngioma, hormone replacement therapy plays a critical role in managing endocrine insufficiency due to pituitary damage, as demonstrated by our case. The patient had long been reliant on hydrocortisone, levothyroxine, and intermittent desmopressin for managing hypopituitarism and diabetes insipidus following her previous craniopharyngioma resection. During pregnancy, the physiological demands on the endocrine system increase significantly, necessitating close monitoring and adjustments to hormone therapy. In this case, hydrocortisone was appropriately increased during times of stress, such as surgery, in line with recommendations for stress-dose glucocorticoids to prevent adrenal crisis. Desmopressin management was critical in addressing DI while maintaining fluid balance, especially given the risk of hypernatremia during pregnancy. Levothyroxine was maintained to ensure proper thyroid function, which is crucial for both maternal and fetal development. The literature highlights the importance of individualized hormone management for pregnant women with hypopituitarism, adjusting for increased metabolic demands in later pregnancy stages and in response to perioperative stress.

## 4 Conclusion

**Figure 4:**
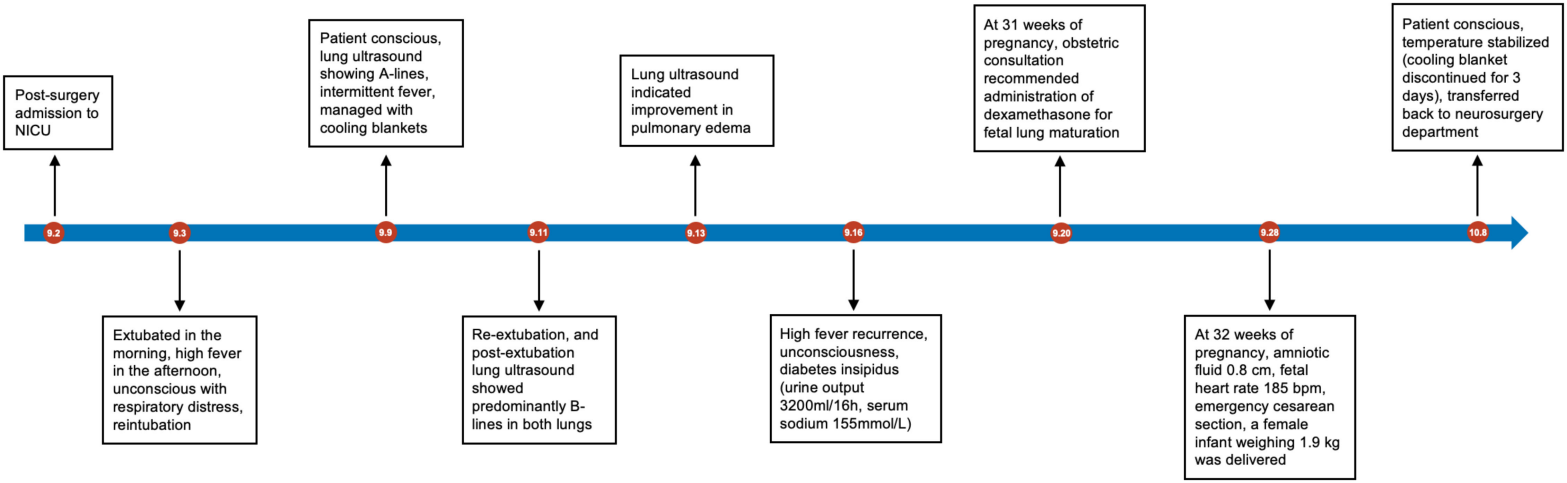
Timeline illustrating key events in the treatment of this case.

We report the case of a 26-year-old woman, 27 weeks pregnant, hospitalized due to a recurrence of craniopharyngioma. Despite the recurrence during pregnancy, the patient chose to continue the pregnancy. An MDT team performed a successful endoscopic transnasal tumor resection, after which the patient’s consciousness improved. At 32 weeks, she delivered a healthy baby girl via cesarean section. We thoroughly discussed the surgical strategy, fluid management, temperature regulation, and hormone replacement therapy throughout this process (**Figure 4**). The medical team maintained open communication with the patient and her family throughout, respecting their wish to continue the pregnancy, and demonstrating patient-centered care. It offers valuable insights into managing pregnant patients with craniopharyngioma, emphasizing the importance of precision medicine and compassionate care to ensure the safety of both mother and child.

## Data Availability

The raw data supporting the conclusions of this article will be made available by the authors, without undue reservation.
