# Supplementary material for: Case report: A pregnant woman with recurrent craniopharyngioma: surgical decision-making and doctor-patient bond
Source: Front Oncol. 2024 Dec 23;14:1508803. doi: 10.3389/fonc.2024.1508803 (PMC11701027; doi:10.3389/fonc.2024.1508803)
Supplement: Supplementary file 1 [file DataSheet1.docx]

**Supplemental Figure 1**


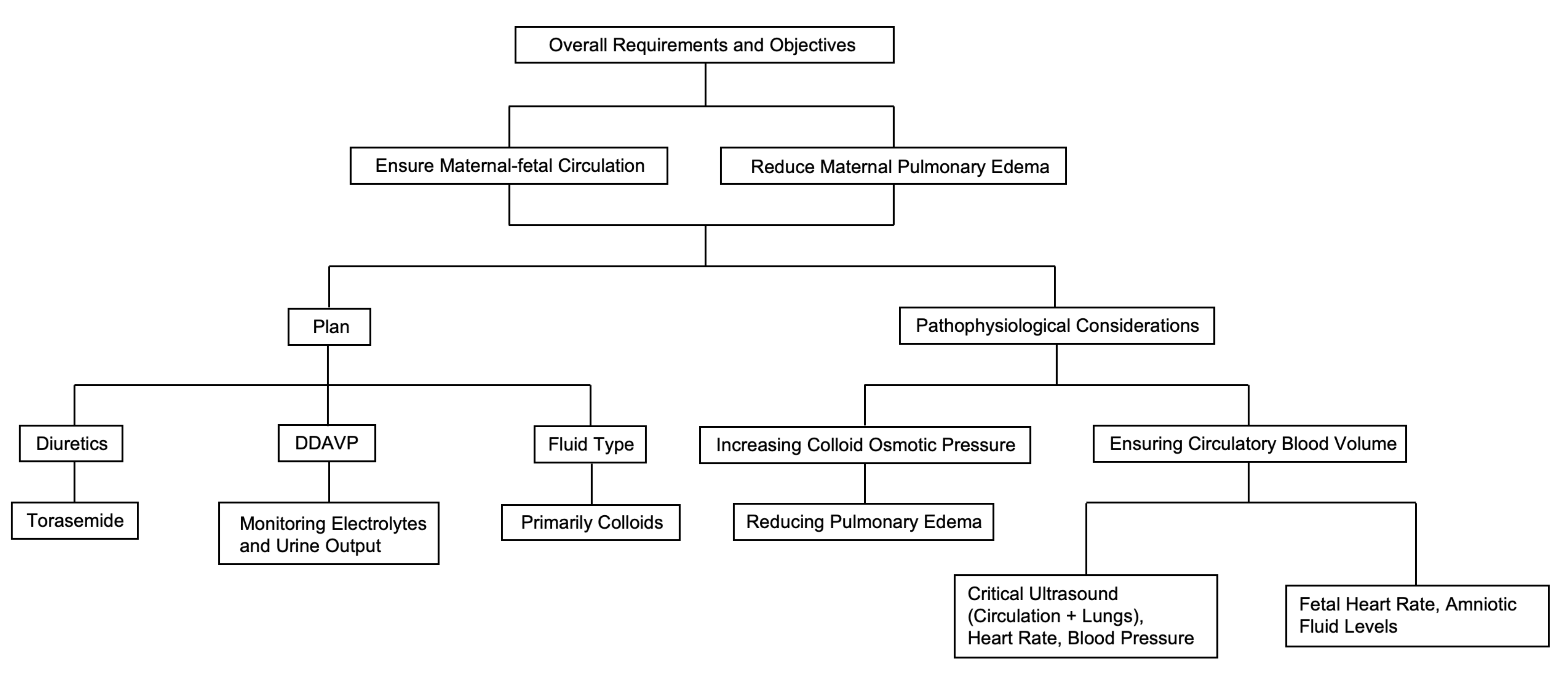


Supplemental Figure 1. The fluid management strategy of this patient focused on reducing maternal pulmonary edema and ensuring maternal-fetal circulation. The overall approach includes a plan involving diuretics (torasemide), desmopressin (DDAVP) with careful monitoring of electrolytes and urine output, and fluid type selection primarily based on colloids to increase colloid osmotic pressure. Pathophysiological considerations emphasize increasing colloid osmotic pressure to reduce pulmonary edema, ensuring circulatory blood volume, and monitoring both maternal and fetal status through critical ultrasound for cardiac and pulmonary assessment, fetal heart rate, and amniotic fluid levels.
